# Scientific basis for standardization of fetal head measurements by ultrasound: a reproducibility study

**DOI:** 10.1002/uog.15956

**Published:** 2016-07-05

**Authors:** R. Napolitano, V. Donadono, E. O. Ohuma, C. L. Knight, S. Z. Wanyonyi, B. Kemp, T. Norris, A. T. Papageorghiou

**Affiliations:** ^1^ Nuffield Department of Obstetrics & GynaecologyUniversity of OxfordOxfordUK; ^2^ Oxford Maternal & Perinatal Health Institute, Green Templeton CollegeUniversity of OxfordOxfordUK; ^3^ Centre for Statistics in Medicine, Botnar Research CentreUniversity of OxfordOxfordUK

**Keywords:** biparietal diameter, fetal head biometry, head circumference, occipitofrontal diameter, reproducibility, transthalamic and transventricular plane, ultrasound, variability

## Abstract

**Objective:**

To compare the standard methods for ultrasound measurement of fetal head circumference (HC) and biparietal diameter (BPD) (outer‐to‐outer (BPDoo) vs outer‐to‐inner (BPDoi) caliper placement), and compare acquisition of these measurements in transthalamic (TT) vs transventricular (TV) planes.

**Methods:**

This study utilized ultrasound images acquired from women participating in the Oxford arm of the INTERGROWTH‐21^st^ Project. In the first phase of the study, BPDoo and BPDoi were measured on stored images. In the second phase, real‐time measurements of BPD, occipitofrontal diameter (OFD) and HC in TT and TV planes were obtained by pairs of sonographers. Reproducibility of measurements made by the same (intraobserver) and by different (interobserver) sonographers, as well as the reproducibility of caliper placement and measurements obtained in different planes, was assessed using Bland–Altman plots.

**Results:**

In Phase I, we analyzed ultrasound images of 108 singleton fetuses. The mean intraobserver and interobserver differences were < 2% (1.34 mm) and the 95% limits of agreement were < 5% (3 mm) for both BPDoo and BPDoi. Neither method for measuring BPD showed consistently better reproducibility. In Phase II, we analyzed ultrasound images of 100 different singleton fetuses. The mean intraobserver and interobserver differences were < 1% (2.26 mm) and the 95% limits of agreement were < 8% (14.45 mm) for all fetal head measurements obtained in TV and TT planes. Neither plane for measuring fetal head showed consistently better reproducibility. Measurement of HC using the ellipse facility was as reproducible as HC calculated from BPD and OFD. OFD by itself was the least reproducible of all fetal head measurements.

**Conclusions:**

Measurements of BPDoi and BPDoo are equally reproducible; however, we believe BPDoo should be used in clinical practice as it allows fetal HC to be measured and compared with neonatal HC. For all head measurements, TV and TT planes provide equally reproducible values at any gestational age, and HC values are similar in both planes. Fetal head measurement in the TT plane is preferable as international standards in this plane are available; however, measurements in the TV plane can be plotted on the same standards. Copyright © 2016 ISUOG. Published by John Wiley & Sons Ltd.

## Abstract 2

Linked Comment: Ultrasound Obstet Gynecol 2016; 48: 26–26

## INTRODUCTION

Fetal head biometry is important for estimation of gestational age in the second trimester and for monitoring fetal growth. Unfortunately, even after decades of clinical practice, guidelines still vary as to how the measurements should be taken, i.e. whether the biparietal diameter (BPD) should be measured by outer‐to‐outer (BPDoo) or outer‐to‐inner (BPDoi) caliper placement^1^, ^2^. It is also uncertain whether head circumference (HC) should be calculated from the occipitofrontal diameter (OFD) and BPD (HC_calculated_) or by using the ellipse facility (HC_ellipse_) on the ultrasound machine, and which is the better plane to use, i.e. transthalamic (TT) or transventricular (TV)^1^, ^3^. These issues are important clinically because measurement inconsistencies may affect the management of individual pregnancies, make it difficult to compare data across units and contribute to the heterogeneity of studies describing fetal size^4^, ^5^.

In this study, we aimed to compare (i) the standard methods for measuring fetal HC (HC_ellipse_ *vs* HC_calculated_) and BPD (BPDoo *vs* BPDoi caliper placement) on ultrasound and (ii) the effect of acquiring head measurements in TT *vs* TV planes, so as to make recommendations regarding best practice.

## SUBJECTS AND METHODS

This study involved women at low risk of adverse pregnancy outcome who were recruited into the Oxford arm of the INTERGROWTH‐21^st^ Project (www.intergrowth21.org.uk), a multicenter, multiethnic, population‐based project, conducted between 2008 and 2014 across eight countries^6^. The Fetal Growth Longitudinal Study (FGLS) is one of the three main components of the INTERGROWTH‐21^st^ Project, which aimed to construct international standards for fetal growth. All women included in our study were part of the FGLS. In the FGLS, serial two‐dimensional ultrasound scans were performed every 5 ± 1 weeks, from 14 + 0 to 41 + 6 weeks' gestation, and images were stored for later analysis. Inclusion criteria for the FGLS were pregnant women with a known, certain last menstrual period, who had regular menstrual cycles and were not taking hormonal contraceptives or breastfeeding in the 2 months before they conceived naturally. Gestational age was calculated using the last menstrual period, with ultrasound confirmation based on a crown–rump length measurement at 9 + 0 to 13 + 6 weeks' gestation that was in agreement by ≤ 7 days^7^, ^8^.

All ultrasound scans in the FGLS were performed by sonographers who were trained, standardized and regularly audited^2^, ^8^, ^9^. At each examination, BPDoo, OFD and HC_ellipse_ were acquired in triplicate in the TT plane. The same commercially available ultrasound machine (Philips HD‐9, Philips Ultrasound, Bothell, WA, USA) with curvilinear abdominal transducers (C5‐2, C6‐3 and V7‐3) was used at all study sites. For the purposes of the INTERGROWTH‐21^st^ Project, the manufacturer reprogrammed the machine's software to ensure that measurement values did not appear on the screen, so as to reduce operator ‘expected value’ bias^2^. The INTERGROWTH‐21^st^ Project was approved by the Oxfordshire Research Ethics Committee ‘C’ (reference: 08/H0606/139) and all participants gave written informed consent.

### Phase I: evaluation of biparietal diameter caliper placement

**Figure 1:**
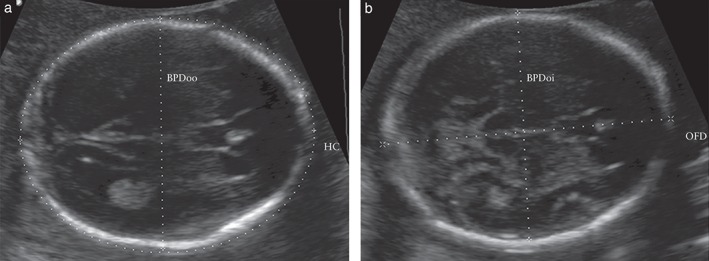
(a) Ultrasound image of biparietal diameter, measured using outer‐to‐outer caliper placement (BPDoo), and fetal head circumference (HC), measured using the ellipse facility, in the transventricular plane. (b) Ultrasound image of biparietal diameter, measured using outer‐to‐inner caliper placement (BPDoi), and occipitofrontal diameter (OFD) in the transthalamic plane.

Using the stored ultrasound images acquired in the FGLS, two sonographers twice measured the BPD using two methods (BPDoo (Figure 1a) and BPDoi (Figure 1b)) on the first of the three images, after the original caliper placements had been removed from the image. The sonographers were blinded to their own and each other's measurements. The intraobserver reproducibility for both methods was calculated for the two sonographers. To calculate the interobserver reproducibility, the first measurements of Sonographer A were compared with those of Sonographer B, and then repeated for the second measurements.

### Phase II: evaluation of transthalamic and transventricular planes

From a cohort of participants that was different from that in Phase I, two sonographers obtained real‐time measurements of BPDoo, OFD and HC_ellipse_ in the TV (Figure  1a) and TT (Figure 1b) planes in duplicate, providing an additional set of images to those in the FGLS. As no difference was found between BPDoo and BPDoi in Phase I, only BPDoo was measured to reduce scanning time. All measurements were obtained in a blinded fashion and were stored on the ultrasound machine and retrieved after completion of the study.

Each sonographer placed the calipers once on each of the four images acquired per participant (i.e. a total of 12 measurements per sonographer for BPDoo, OFD and HC_ellipse_). Sonographer B repeated the caliper placements on the images acquired by Sonographer A, resulting in a total of 36 measurements. HC was also calculated from BPD and OFD (HC_calculated_) for each image.

### Measurement and plane definitions

BPDoo was measured with the intersection of the calipers placed from the outer edge of the proximal calvarial wall to the outer edge of the distal calvarial wall, at the widest part of the skull (Figure 1a). BPDoi was measured with the intersection of the calipers placed from the outer edge of the proximal calvarial wall to the inner edge of the distal calvarial wall (Figure 1b)^10^. OFD was measured with the intersection of the calipers placed from the outer edge of the anterior frontal wall to the outer edge of the distal occipital wall, at the longest part of the skull (Figure  1b). HC_ellipse_ was measured using the ellipse facility, placing the line of the ellipse on the outer border of the skull (Figure  1b)^2^. The TT plane was acquired according to the following conditions: axial view at the level of the thalami with an angle of insonation as close as possible to 90°; the head had to be oval in shape, symmetrical, centrally positioned and filling at least 30% of the monitor; the midline echo (representing the falx cerebri) had to be broken anteriorly, at a third of its length, by the cavum septi pellucidi; and the thalami had to be located symmetrically on either side of the midline (Figure  1b)^2^. The TV plane was acquired including all the standard parameters to obtain a TT plane but visualizing the lateral ventricles rather than the thalami at a more cranial level, with the ventricles located symmetrically on each side of the midline, the anterior and posterior horns both visible, and the posterior ventricle cavity visualized as a hypoechoic structure (Figure  1a)^1^.

### Statistical analysis

In Phase I, the following analyses were performed: (i) intraobserver reproducibility of caliper placement for measurement of BPD using the BPDoo and BPDoi method, calculated for Sonographers A and B; and (ii) interobserver reproducibility of caliper placement for measurements of BPD using the BPDoo and BPDoi method, comparing the first measurements of Sonographer A with those of Sonographer B, and the second measurements of Sonographer A with those first obtained by Sonographer B.

In Phase II, the following analyses were performed: (i) intraobserver reproducibility of plane acquisition and caliper placement for TT and TV planes, comparing each sonographer's first and second measurements in the same plane; (ii) interobserver reproducibility of plane acquisition and caliper placement for TT and TV planes, comparing measurements of Sonographers A and B in the same plane; (iii) caliper replacement reproducibility, based on Sonographer B replacing the calipers on the images acquired by Sonographer A in the TT and TV planes (interobserver reproducibility); (iv) intraobserver reproducibility of plane acquisition and caliper placement between TT and TV planes, comparing the measurements of Sonographer A acquired in the TT plane with those acquired by Sonographer A in the TV plane (the same was then calculated for Sonographer B); and (v) interobserver reproducibility for plane acquisition and caliper placement between TT and TV planes, comparing the measurements of Sonographer A acquired in the TT plane with those acquired by Sonographer B in the TV plane, and then the measurements of Sonographer B acquired in the TT plane with those acquired by Sonographer A in the TV plane.

Intraobserver and interobserver variability were expressed as a percentage to account for increasing fetal head size with gestational age. Percentages were calculated as the difference between two measurements divided by the average of the two measurements, multiplied by 100. Reproducibility was assessed using Bland–Altman plots. All plots and analyses were performed using STATA 11 (StataCorp, College Station, TX, USA).

Paired or unpaired *t*‐tests, as appropriate, were performed to assess mean differences between measurements obtained by the same sonographer (intraobserver reproducibility) and different sonographers (interobserver reproducibility), and those obtained in two different planes (between‐plane reproducibility). A *P*‐value of < 0.05 was considered statistically significant.

## RESULTS

Four women were included in the study at each gestational week, from 15 to 41 weeks in Phase I (108 women) and from 16 to 40 weeks in Phase II (100 women), resulting in a total of 4464 measurements. The demographic characteristics of the 208 participants are shown in Table  1.

**Table 1:** Demographic characteristics of women with singleton pregnancy recruited into the Fetal Growth Longitudinal Study of the INTERGROWTH‐21^st^ Project who had retrospective measurement of biparietal diameter (BPD) (Phase I) or real‐time measurements of fetal biometry in transthalamic (TT) and transventricular (TV) planes (Phase II)

Characteristic	Phase I: BPD study (*n* = 108)	Phase II: TT/TV study (*n* = 100)
Maternal age (years)	30 ± 4	30 ± 5
BMI (kg/m^2^)	23.3 ± 2.7	26.9 ± 3.9
Nulliparous	66 (61)	42 (42)
GA at scan (weeks)	28.1 ± 7.7	28.0 ± 7.2

Data are given as mean ± SD or *n* (%). BMI, body mass index; GA, gestational age.

### Phase I: evaluation of biparietal diameter caliper placement

A total of 864 measurements were obtained in Phase I. Intraobserver and interobserver reproducibility was very good overall. The mean differences were < 2% (1.34 mm) and the 95% limits of agreement were < 5% (3 mm) for both BPDoo and BPDoi (Table  2 and Figures S1 and S2); however, neither method showed consistently better reproducibility. As expected, the 95% limits of agreement for interobserver reproducibility of BPDoo and BPDoi (3.1–4.2%) were slightly wider than for the intraobserver reproducibility (1.3–2.1%).

**Table 2:** Intra‐ and interobserver reproducibility of biparietal diameter measurement using outer‐to‐outer (BPDoo) or outer‐to‐inner (BPDoi) caliper placement method

Measurement	Intraobserver		Interobserver
	Sonographer A	Sonographer B	
BPDoo	0.01 (2.08)	0.02 (1.28)	1.93 (4.16)
BPDoi	−0.16 (1.63)	−0.15 (1.33)	0.80 (3.10)

Data are given as mean difference (95% limits of agreement (LOA)) in percent. Upper and lower 95% LOA in each case can be calculated as mean difference ± value displayed.

### Phase II: evaluation of transthalamic vs transventricular plane

A total of 3600 measurements (1200 for BPD, OFD and HC_ellipse_) were obtained in Phase II. HC_ellipse_ was marginally larger, by 0.09% (0.61 mm, *P* = 0.034), when measured in the TV than when measured in the TT plane. However, no such difference was observed for BPD or OFD. In terms of overall reproducibility, the mean differences in fetal head measurements were < 1% (2.26 mm) and the 95% limits of agreement were < 8% (14.45 mm) for both TV and TT planes (Figures S3–S7).

Overall, the reproducibility of caliper placement accounted for 50–60% of the reproducibility of measurements obtained in each plane. For example, the 95% limits of agreement for interobserver reproducibility of HC_ellipse_ in the TV plane was 4.87% (Table  3 and Figure S4) and the respective value for reproducibility of caliper replacements in the same plane was 3.05% (Table  3 and Figure S5), constituting approximately 60% of the total reproducibility.

**Table 3:** Intraobserver and interobserver reproducibility of ultrasound measurements of fetal head biometry and caliper replacement in the same plane and between planes

Measurement	Within‐plane reproducibility						Between‐plane reproducibility	
	Intraobserver		Interobserver		Caliper replacement		TT vs TV	
	TT	TV	TT	TV	TT interobserver	TV interobserver	Intraobserver	Interobserver
BPDoo	−0.14 (4.05)	−0.02 (3.43)	0.70 (6.65)	0.09 (4.78)	0.30 (3.16)	0.41 (2.69)	0.24 (5.63)	0.24 (5.84)
OFD	−0.31 (6.55)	−0.41 (5.50)	−0.03 (7.98)	−0.13 (7.66)	0.50 (4.63)	0.86 (4.58)	−0.13 (6.69)	−0.14 (8.11)
HC_ellipse_	−0.06 (3.47)	−0.25 (3.32)	−0.48 (4.78)	−0.75 (4.87)	−0.43 (3.14)	0.12 (3.05)	−0.09 (4.53)	−0.10 (5.11)
HC_calculated_	−0.23 (4.13)	−0.24 (3.53)	0.29 (5.54)	0.02 (5.02)	0.43 (2.91)	0.66 (2.92)	0.04 (4.78)	0.03 (5.50)

Data are given as mean difference (95% limits of agreement (LOA)) in percent. Upper and lower 95% LOA in each case can be calculated as mean difference ± value displayed. BPDoo, biparietal diameter measured using outer‐to‐outer caliper placement; HC_calculated_, head circumference calculated from biparietal diameter and occiptofrontal diameter (OFD); HC_ellipse_, head circumference measured using ellipse facility on ultrasound machine; TT, transthalamic; TV, transventricular.

Neither the TV or TT plane was associated with consistently better reproducibility. In addition, the 95% limits of agreement between sonographers measuring in the same plane (interobserver reproducibility within the same plane) were only slightly wider than the limits of agreement between TV and TT planes acquired and measured by the same sonographer (intraobserver reproducibility between TT and TV planes). This suggests that the effect of two sonographers measuring in the same plane is similar to that of the same sonographer measuring in different planes. The 95% limits of agreement were highest when two sonographers measured in different planes (interobserver reproducibility between TT and TV planes) (Table  3 and Figure S7). Lastly, there was no significant difference between HC_ellipse_ measurements and an equal number of HC_calculated_ measurements.

## DISCUSSION

### Main findings

The aim of this study was to determine the most reproducible method for performing fetal head biometry for clinical practice and research, such as the production of standards. There are two approaches that could have been used. The first is to assess the accuracy of the ultrasound measurements against a ‘gold standard’^11^. However, defining a gold standard for fetal measurements is difficult. For example, magnetic resonance imaging allows clear visualization of the fetus, but estimates are still associated with errors^12^. The use of phantoms has obvious limitations as inanimate structures do not effectively represent the variability of live structures^13^. The second approach is to assess the reproducibility of different methods of measuring fetal head biometry and to use the one with least error and bias^14^.

We found no major differences in the reproducibility of caliper placement for measuring BPDoo or BPDoi. Similarly, there was no difference in the reproducibility of measuring HC in the TV or TT planes. Using the ellipse facility (HC_ellipse_) to measure HC was marginally more reproducible than using the two‐diameters method (HC_calculated_), with the former having interobserver 95% limits of agreement of just below 5% and the latter having interobserver 95% limits of agreement of just above 5%. This is probably due to the contribution of the OFD, which is the least reproducible head measurement in the two‐diameters method.

The BPDoi method was used originally because the inner margin of the fetal skull in the distal field was sharper when using static B scanners^15^, ^16^, ^17^, ^18^. However, modern equipment produces a clearer image and so the BPDoi method appears to have no measurable effect on reproducibility (Table  2), even though caliper replacement constitutes up to 60% of the total variability. Therefore, choosing between BPDoo and BPDoi should be for reasons other than trying to reduce error, such as the protocol used (BPDoo) to develop international standards for monitoring fetal growth^19^. Another reason for using BPDoo is that it enables direct comparisons to be made between antenatal and postnatal measurements of HC^20^, ^21^.

Lastly, neither the TV nor TT plane was found to be consistently associated with better reproducibility. We did find that biometry in the TV plane yielded a very slightly larger HC than that measured in the TT plane. Although this was statistically significant, it was not clinically relevant (< 0.1%, 0.61 mm). Furthermore, when comparing the reproducibility of measuring HC in the TT and TV planes, the difference between sonographers measuring in the same plane was similar to that of the same sonographer measuring in different planes.

### Limitations and strengths

There are some limitations to our study. It can be argued that the use of six different sonographers working in pairs (rather than one pair) might have had an impact on the results. However, we feel that the study design more accurately reflects clinical practice, as most units have several qualified sonographers^22^. The setting of near‐optimal conditions (i.e. experienced sonographers, healthy population and a scientifically rigorous study design) may be seen as creating an artificial setting. However, such conditions were necessary to minimize the contribution of confounding factors so as to define the variability in relation to the research question as purely as possible, which we see as a strength. The other strengths of our study were that reproducibility was assessed throughout pregnancy by recruiting a fixed number of women per week of gestation, and recommended methods^23^ were used that have been shown to be the most appropriate for assessing the reproducibility of two measurements^24^, ^25^.

### Our findings in context with other studies

A literature search was performed to identify all publications reporting reproducibility in the evaluation of fetal head biometry. We searched MEDLINE using the following keywords: biparietal diameter OR BPD OR occipitofrontal diameter OR OFD OR head circumference OR HC AND fetal OR foetal OR fetus OR foetus AND ultrasound OR ultrasonogra* OR ultra‐sonogra* OR sonic* OR scan* AND reproducibility OR variability OR repeatability. Restrictions that were applied were studies in humans, in the English language and published after 1970. Additional references were added from an important article^4^. Nineteen relevant studies were identified (Table S1)^15^, ^16^, ^17^, ^18^, ^22^, ^26^, ^27^, ^28^, ^29^, ^30^, ^31^, ^32^, ^33^, ^34^, ^35^, ^36^, ^37^, ^38^, ^39^. In most, the primary aim of the study was not to assess reproducibility but to build growth charts. The studies reporting either BPD method did not reveal large differences from our findings (the reported mean differences were < 2% for BPDoi, with limits of agreement of < 5%^15^, ^16^, ^17^, ^18^, ^34^, ^36^, and there were only two small studies^29^, ^38^ on BPDoo showing limits of agreement of 3.8 and 7.4 mm, respectively). In only one study was the reproducibility of BPDoo and BPDoi reported in the same group of fetuses, which showed repeatability coefficients that were similar for both methods^34^. Measurements of HC_ellipse_ were reproducible, with a mean difference of 3.5 mm and limits of agreement of < 12 mm (5%), in line with our results^15^, ^16^, ^17^, ^22^, ^27^, ^28^, ^29^, ^34^, ^35^, ^39^. No previous study was found comparing the two different planes of acquisition (TV *vs* TT) in the same population.

In conclusion, using modern ultrasound equipment, measurement of BPD is equally reproducible irrespective of whether calipers are placed BPDoo or BPDoi. However, BPDoo can be used for both BPD and HC measurements and is also the method to measure OFD. It therefore seems simplest to use BPDoo as a conceptually similar methodological approach for all head measurements. BPDoo is also clinically useful (as part of the HC_calculated_) for monitoring growth from the ‘womb to the classroom’^40^, as it is possible to track head size and growth from the antenatal to postnatal periods^41^. We found that HC measurements using HC_ellipse_ were associated with slightly better interobserver reproducibility than using HC_calculated_, based on BPD and OFD. However, there was no large difference in reproducibility of BPD, OFD or HC_ellipse_ measured in the TV compared with TT plane. The mean difference in head size between these two planes was also minimal (< 1%) at every gestational age.

We therefore recommend that standard fetal head biometry measurements are performed using the BPDoo, OFD and HC_ellipse_, all measured in the TT plane, based on the reproducibility evidence presented in this study and the existence of international standards based on these methods. In centers in which HC is measured in the TV plane, use of the international standards is still appropriate^19^.

## Supporting information


**Table S1** and **Figures S1–S7** may be found in the online version of this article.
