# Supplementary material for: Scientific basis for standardization of fetal head measurements by ultrasound: a reproducibility study
Source: Ultrasound Obstet Gynecol. 2016 Jul 5;48(1):80–5. doi: 10.1002/uog.15956 (PMC5113683; doi:10.1002/uog.15956)
Supplement: Supplementary file 1 — Table S1 and Figures S1–S7 may be found in the online version of this article. [file UOG-48-80-s001.zip › Figures S1 and S2.docx]

**Figure S1** Bland–Altman plots showing intraobserver reproducibility for Sonographers A and B of outer-to-outer and outer-to-inner caliper placement when measuring biparietal diameter (BPD) in the transthalamic plane. Plots on left show absolute difference (in mm) and plots on right show reproducbility as a percentage.

Intraobserver reproducibility for BPD outer to outer (%) – Sonographer A

Intraobserver reproducibility for BPD outer to outer (mm) – Sonographer A

Intraobserver reproducibility for BPD outer to outer (mm) – Sonographer B

Intraobserver reproducibility for BPD outer to outer (%) – Sonographer B

Intraobserver reproducibility for BPD outer to inner (%) – Sonographer A

Intraobserver reproducibility for BPD outer to inner (mm) – Sonographer A

Intraobserver reproducibility for BPD outer to inner (%) – Sonographer B

Intraobserver reproducibility for BPD outer to inner (mm) – Sonographer B

**Figure S2** Bland–Altman plots showing interobserver reproducibility of outer-to-outer and outer-to-inner caliper placement when measuring biparietal diameter (BPD) in the transthalamic plane. Plots on left show absolute difference (in mm) and plots on right show reproducbility as a percentage.

Interobserver reproducibility for BPD outer to outer (mm)

Interobserver reproducibility for BPD outer to outer (%)

Interobserver reproducibility for BPD outer to inner (mm)

Interobserver reproducibility for BPD outer to inner (%)

Interobserver reproducibility for BPD outer to inner (mm)
