# Supplementary material for: Scientific basis for standardization of fetal head measurements by ultrasound: a reproducibility study
Source: Ultrasound Obstet Gynecol. 2016 Jul 5;48(1):80–5. doi: 10.1002/uog.15956 (PMC5113683; doi:10.1002/uog.15956)
Supplement: Supplementary file 1 — Table S1 and Figures S1–S7 may be found in the online version of this article. [file UOG-48-80-s001.zip › Figures S3-S7.docx]

**Figure S3** Bland–Altman plots showing intraobserver reproducibility, in the transthalamic and transventricular planes, of acquiring and measuring head circumference using the ellipse facility (HC_ellipse_)_,_ biparietal diameter (BPD), occipitofrontal diameter (OFD) and head circumference calculated from the two perpendicular head diameters BPD and OFD (HC_calculated_). Plots on left show absolute difference (in mm) and plots on right show reproducbility as a percentage.

Intraobserver reproducibility for HC_ellipse_

Transthalamic plane (mm)

Intraobserver reproducibility for HC_ellipse_

Transthalamic plane (%)

Intraobserver reproducibility for HC_ellipse_

Transventricular plane (%)

Intraobserver reproducibility for HC_ellipse_

Transvetricular plane (mm)

Intraobserver reproducibility for BPD

Transthalamic plane (%)

Intraobserver reproducibility for BPD

Transthalamic plane (mm)

Intraobserver reproducibility for BPD

Transventricular plane (%)

Intraobserver reproducibility for BPD

Transventricular plane (mm)

Intraobserver reproducibility for OFD

Transthalamic plane (mm)

Intraobserver reproducibility for OFD

Transthalamic plane (%)

Intraobserver reproducibility for OFD

Transventricular plane (mm)

Intraobserver reproducibility for OFD

Transventricular plane (%)

Intraobserver reproducibility for HC_calculated_

Transthalamic plane (%)

Intraobserver reproducibility for HC_calculated_

Transthalamic plane (mm)

Intraobserver reproducibility for HC_calculated_

Transventricular plane (%)

Intraobserver reproducibility for HC_calculated_

Transvetricular plane (mm)

**Figure S4** Bland–Altman plots showing interobserver reproducibility, in the transthalamic and transventricular planes, of acquiring and measuring head circumference using the ellipse facility (HC_ellipse_)_,_ biparietal diameter (BPD), occipitofrontal diameter (OFD) and head circumference calculated from the two perpendicular head diameters BPD and OFD (HC_calculated_). Plots on left show absolute difference (in mm) and plots on right show reproducbility as a percentage.

Interobserver reproducibility for HC_ellipse_

Transthalamic plane (%)

Interobserver reproducibility for HC_ellipse_

Transthalamic plane (mm)

Interobserver reproducibility for HC_ellipse_

Transvetricular plane (mm)

Interobserver reproducibility for HC_ellipse_

Transventricular plane (%)

Interobserver reproducibility for BPD

Transthalamic plane (mm)

Interobserver reproducibility for BPD

Transthalamic plane (%)

Interobserver reproducibility for BPD

Transventricular plane (%)

Interobserver reproducibility for BPD

Transventricular plane (mm)

Interobserver reproducibility for BPD

Transventricular plane (mm)

Interobserver reproducibility for BPD

Transventricular plane (%)

Interobserver reproducibility for OFD

Transthalamic plane (%)

Interobserver reproducibility for OFD

Transthalamic plane (mm)

Interobserver reproducibility for OFD

Transventricular plane (mm)

Interobserver reproducibility for OFD

Transventricular plane (%)

Interobserver reproducibility for HC_calculated_

Transthalamic plane (%)

Interobserver reproducibility for HC_calculated_

Transthalamic plane (mm)

Interobserver reproducibility for HC_calculated_

Transventricular plane (%)

Interobserver reproducibility for HC_calculated_

Transvetricular plane (mm)

**Figure S5** Bland–Altman plots showing interobserver reproducibility of caliper replacement, in transthalamic and transventricular planes, for measuring head circumference using the ellipse facility (HC_ellipse_), biparietal diameter (BPD), occipitofrontal diameter (OFD) and head circumference calculated from the two perpendicular head diameters BPD and OFD (HC_calculated_). Plots on left show absolute difference (in mm) and plots on right show reproducbility as a percentage.

Interobserver reproducibility for HC_ellipse_

Transthalamic plane (%)

Interobserver reproducibility for HC_ellipse_

Transthalamic plane (mm)

Interobserver reproducibility for HC_ellipse_

Transventricular plane (%)

Interobserver reproducibility for HC_ellipse_

Transvetricular plane (mm)

Interobserver reproducibility for BPD

Transthalamic plane (mm)

Interobserver reproducibility for BPD

Transthalamic plane (%)

Interobserver reproducibility for BPD

Transventricular plane (mm)

Interobserver reproducibility for BPD

Transventricular plane (%)

Interobserver reproducibility for OFD

Transthalamic plane (%)

Interobserver reproducibility for OFD

Transthalamic plane (mm)

Interobserver reproducibility for OFD

Transventricular plane (mm)

Interobserver reproducibility for OFD

Transventricular plane (%)

Interobserver reproducibility for HC_calculated_

Transthalamic plane (mm)

Interobserver reproducibility for HC_calculated_

Transthalamic plane (%)

Interobserver reproducibility for HC_calculated_

Transvetricular plane (mm)

Interobserver reproducibility for HC_calculated_

Transventricular plane (%)

**Figure S6** Bland–Altman plots showing between-plane intraobserver reproducibility in the transthalamic and transventricular planes, of acquiring and measuring the head circumference using the ellipse facility (HC_ellipse_), biparietal diameter (BPD), occipitofrontal diameter (OFD), head circumference calculated from the two perpendicular head diameters (HC_calculated_). Plots on left show absolute difference (in mm) and plots on right show reproducbility as a percentage.

Between planes intraobserver reproducibility for HC_ellipse_ (mm)

() of HC

Between planes intraobserver reproducibility for HC_ellipse_ (%)

Between planes intraobserver reproducibility for BPD (mm)

Between planes intraobserver reproducibility for BPD (%)

Between planes intraobserver reproducibility for OFD (%)

Between planes intraobserver reproducibility for OFD (mm)

Between planes intraobserver reproducibility for HC_calculated_ (%)

Between planes intraobserver reproducibility for HC_calculated_ (mm)

**Figure S7** Bland–Altman plots showing between-plane interobserver reproducibility in transthalamic and transventricular planes, of acquiring and measuring head circumference using the ellipse facility (HC_ellipse_), biparietal diameter (BPD), occipitofrontal diameter (OFD), head circumference calculated from the two perpendicular head diameters BPD and OFD (HC_calculated_). Plots on left show absolute difference (in mm) and plots on right show reproducbility as a percentage.

Between planes interobserver reproducibility for HC_ellipse_ (mm)

Between planes interobserver reproducibility for HC_ellipse_ (%)

Between planes interobserver reproducibility for BPD (mm)

Between planes interobserver reproducibility for BPD (%)

Between planes interobserver reproducibility for OFD (%)

Between planes interobserver reproducibility for OFD (mm)

Between planes interobserver reproducibility for HC_calculated_ (%)

Between planes interobserver reproducibility for HC_calculated_ (mm)
