# Supplementary material for: Scientific basis for standardization of fetal head measurements by ultrasound: a reproducibility study
Source: Ultrasound Obstet Gynecol. 2016 Jul 5;48(1):80–5. doi: 10.1002/uog.15956 (PMC5113683; doi:10.1002/uog.15956)
Supplement: Supplementary file 1 — Table S1 and Figures S1–S7 may be found in the online version of this article. [file UOG-48-80-s001.zip › Table S1.docx]

**Table S1** Studies reporting on quantitative reproducibility of fetal head biometry identified in literature search

| Reference | *n* | GA (weeks) | Biometry measured | Plane | BA plots reported | BPD mean difference (mm (%)) | | BPD 95% CI (mm (%)) | | HC mean difference (mm (%)) | | HC 95% CI (mm (%)) | |
| --- | --- | --- | --- | --- | --- | --- | --- | --- | --- | --- | --- | --- | --- |
|  |  |  |  |  |  | Intra. | Intra. | Inter. | Inter. | Intra. | Inter. | Intra. | Inter. |
| [Al-Meshari (1987](file:///E:\Dropbox\TT%20vs%20TV\TV%20vs%20TT\ARTICLES!!!\AlMeshari.pdf)) | 100 | 13–36 | BPDoi, HC, OFD | TT | No | 1.8 | 1.0 | 1.0 |  |  |  | 1.0 | 1.0 |
| [Bergsjo](file:///E:\Dropbox\TT%20vs%20TV\TV%20vs%20TT\ARTICLES!!!\Bergsjo%201975%20actObsGynScand%20observ%20error%20head.pdf) (1976) | 71 | 26–44 | BPD | NA | No |  |  | 3.04 |  |  |  |  |  |
| [Chan (2009](file:///E:\Dropbox\TT%20vs%20TV\TV%20vs%20TT\ARTICLES!!!\Chan%202009%20UOG%202d%20vs%203d.pdf)) | 36 | 22–30 | BPDoi, HC | TT | Yes |  | 1.3 | 1.7 |  |  |  | 8.8 | 8.1 |
| [Deter](file:///E:\Dropbox\TT%20vs%20TV\TV%20vs%20TT\ARTICLES!!!\Deter%201982%20j%20clin%20ultras%20head%20and%20abdomen.pdf) (1982) | 110 | 18–34 | HC | NA | No |  |  |  |  | –0.69 |  | 2.02 |  |
| [Di Battista (2000](file:///E:\Dropbox\TT%20vs%20TV\TV%20vs%20TT\ARTICLES!!!\Di%20Battista2000.pdf))* | 20 | 13–36 | BPDoi, HC, OFD | TT | No |  |  |  | 1T: 0.67  3T: 0.76 |  | 2T: 2.43  3T: 3.49 |  |  |
| [Gull (2002](file:///E:\Dropbox\TT%20vs%20TV\TV%20vs%20TT\ARTICLES!!!\Gull%20UOG%202002%20intervariability%20BPD%20HC%20EFW.pdf)) | 39 | 38–41 | BPDoi, HC | TT | No | 1.42 (1.88) | 2.4 (3.5) |  |  |  |  | 3.4 (4.3) |  |
| [Hadlock](file:///E:\Dropbox\TT%20vs%20TV\TV%20vs%20TT\ARTICLES!!!\hadlock1982b.pdf) (1982) | 26 | 15–41 | HC | NA | No |  |  |  |  | CP: 0.3 Ellipse: 0.3 |  | CP: 7.8  Ellipse: 11.2 |  |
| [Johnsen (2006](file:///E:\Dropbox\TT%20vs%20TV\TV%20vs%20TT\ARTICLES!!!\johnsen2006.pdf)) | 20 | 12–31 | BPDoo, HC | TT | No |  | 3.8 | 3.8 |  |  |  | 11.8 | 11.8 |
| [Krampl (2000](file:///E:\Dropbox\TT%20vs%20TV\TV%20vs%20TT\ARTICLES!!!\krampl%202002%20UOG%20fetal%20biometry.pdf)) | 62 | 14–42 | BPDoo, HC, OFD | TV | Yes |  |  |  |  |  |  |  |  |
| [Larsen (1990](file:///E:\Dropbox\TT%20vs%20TV\TV%20vs%20TT\ARTICLES!!!\larsen1990.pdf)) | 5 | 27–38 | BPDoi, HC | TV | No | 0.95 |  |  |  |  |  |  |  |
| [Lima (2012](file:///E:\Dropbox\TT%20vs%20TV\TV%20vs%20TT\ARTICLES!!!\Lima%202012%20UOG.pdf)) | 102 | 24–40 | BPDoi | TT | Yes |  | 2.9 (4.01) | 3.4 (4.6) |  |  |  |  |  |
| [Merialdi (2005](file:///E:\Dropbox\TT%20vs%20TV\TV%20vs%20TT\ARTICLES!!!\Merialdi%20UOG%202005%20Perun%20oo.pdf)) | NA | 24–38 | BPDoo, HC | NA | No |  |  |  |  |  |  |  |  |
| [Pang (2003](file:///E:\Dropbox\TT%20vs%20TV\TV%20vs%20TT\ARTICLES!!!\pang2003.pdf)) | NA | 24–40 | BPDoi, BPDoo, HC | TT | No |  |  | 1.4†; 1.95‡ |  |  |  |  | 1.97 |
| [Perni (2004](file:///E:\Dropbox\TT%20vs%20TV\TV%20vs%20TT\ARTICLES!!!\Perni%202004%20UOG.pdf)) | 122 | 15–40 | BPD, HC | TT | Yes | 0.2 | 2.5 | 2.8 | 0.2 | 0.6 | 0.1 | 9.3 | 10.9 |
| [Persson (1978](file:///E:\Dropbox\TT%20vs%20TV\TV%20vs%20TT\ARTICLES!!!\person1978.pdf)) | 30 | 16–40 | BPDoi | NA | No |  |  | 1.8 | Same day: 0.05  Different day: 0.44 |  |  |  |  |
| [Salpou (2008](file:///E:\Dropbox\TT%20vs%20TV\TV%20vs%20TT\ARTICLES!!!\Salpou%20Bio%20Med%20Centr%202008%20africa.pdf)) | 200 | 12–22 | BPDoi, BPDoo, HC | TT | No |  |  |  |  |  |  |  |  |
| [Sarris (2012](file:///E:\Dropbox\TT%20vs%20TV\TV%20vs%20TT\ARTICLES!!!\sarris%202012%20UOG.pdf)) | 140 | 14–41 | BPDoo, HC, OFD | TT | Yes |  |  |  |  |  |  | CP: 4.5 (2.4)  Ellipse: 7.0 (3.0)  Calculated: 7.2 (3.1) | CP: 9.8 (3.7)  Ellipse: 12.1 (4.9)  Calculated: 12.0 (4.9) |
| [Shepard (1982](file:///E:\Dropbox\TT%20vs%20TV\TV%20vs%20TT\ARTICLES!!!\Shepard%201982%20JUM%20BPD%20plane.pdf)) | 18 | NA | BPDoo | TT | No |  |  | 7.4 |  |  |  |  |  |
| [Yang (2010](file:///E:\Dropbox\TT%20vs%20TV\TV%20vs%20TT\ARTICLES!!!\Yang%202010%20UOG.pdf)) | 50 | 17–34 | BPD, HC | NA | Yes | Op1: -0.09 Op2: 0.16 |  |  | -0.09 | Op1: -0.27 Op 2: -0.2 | 0.66 |  |  |

Only the first author of each study is given. *Only study reporting occipitofrontal diameter (OFD) mean difference in intraobserver reproducibility in the second trimester (2T) (1.26 mm) and third trimester (3T) (1.46 mm). †Biparietal diameter outer-to-inner (BPDoi). ‡Biparietal diameter outer-to-outer (BPDoo). 1T, first trimester; BA, Bland–Altman plot; BPD, biparietal diameter; CP, caliper placement; GA, gestational age; HC, head circumference; Inter., interobserver reproducibility; Intra., intraobserver reproducibility; NA, not available; Op, operator; TT, transthalamic plane; TV, transventricular plane.
